# Uncharged Components of Single-Stranded DNA Modulate Liquid–Liquid Phase Separation With Cationic Linker Histone H1

**DOI:** 10.3389/fcell.2021.710729

**Published:** 2021-08-04

**Authors:** Masahiro Mimura, Shunsuke Tomita, Hiroka Sugai, Yoichi Shinkai, Sayaka Ishihara, Ryoji Kurita

**Affiliations:** ^1^Faculty of Pure and Applied Sciences, University of Tsukuba, Tsukuba, Japan; ^2^Health and Medical Research Institute, National Institute of Advanced Industrial Science and Technology (AIST), Tsukuba, Japan; ^3^Biomedical Research Institute, National Institute of Advanced Industrial Science and Technology (AIST), Tsukuba, Japan

**Keywords:** phase separation, intrinsically disordered proteins, liquid droplets, DNA, linker histone H1

## Abstract

Liquid–liquid phase separation (LLPS) of proteins and DNAs has been recognized as a fundamental mechanism for the formation of intracellular biomolecular condensates. Here, we show the role of the constituent DNA components, i.e., the phosphate groups, deoxyribose sugars, and nucleobases, in LLPS with a polycationic peptide, linker histone H1, a known key regulator of chromatin condensation. A comparison of the phase behavior of mixtures of H1 and single-stranded DNA-based oligomers in which one or more of the constituent moieties of DNA were removed demonstrated that not only the electrostatic interactions between the anionic phosphate groups of the oligomers and the cationic residues of H1, but also the interactions involving nucleobases and deoxyriboses (i) promoted the generation of spherical liquid droplets via LLPS as well as (ii) increased the density of DNA and decreased its fluidity within the droplets under low-salt conditions. Furthermore, we found the formation of non-spherical assemblies with both mobile and immobile fractions at relatively higher concentrations of H1 for all the oligomers. The roles of the DNA components that promote phase separation and modulate droplet characteristics revealed in this study will facilitate our understanding of the formation processes of the various biomolecular condensates containing nucleic acids, such as chromatin organization.

## Introduction

Liquid–liquid phase separation (LLPS) triggered by interactions between biomacromolecules has increasingly been recognized as a possible mechanism governing the formation of biomolecular condensates, such as germline P granules (Brangwynne et al., 2009) and stress granules (Boeynaems et al., 2018). These condensates are often referred to as ‘liquid droplets’ due to their fluidic properties (Brangwynne et al., 2009; Uversky, 2017) and are distinct from the solid-like aggregates (Patel et al., 2015). The fluidic properties of liquid droplets enable their reversible and rapid formation in response to various endogenous and exogenous changes in the intracellular environment, as well as the selective concentration or exclusion of particular biomolecules within the droplet phase and the dynamic exchange of biomolecules with the surrounding phase (Nakashima et al., 2019). Therefore, liquid droplets are likely to serve as transient reaction compartments for a wide range of biological processes (Yoshizawa et al., 2020; Kamimura and Kanai, 2021).

Recently, biomolecular condensates between proteins and nucleic acids formed via LLPS have been proposed to be associated not only with the regulation of gene transcription (Hnisz et al., 2017), DNA replication (Parker et al., 2019), and DNA damage repair (Laflamme and Mekhail, 2020), but also with the organization of specific chromatin domains (Erdel and Rippe, 2018; Larson and Narlikar, 2018; Gibson et al., 2019). Although the major factor in the formation of these condensates is the LLPS-prone proteins, the DNA itself is also critical in this event. For example, the droplet formation of heterochromatin protein HP1α is promoted by non-specific electrostatic interactions with DNA (Larson et al., 2017). DNA binding is also essential for LLPS of the transcriptional repressor VRN1 (Zhou et al., 2019) and the chromatin component histone proteins (Gibson et al., 2019; Shakya et al., 2020). In many of these examples, the interactions of DNA with nonstructural cationic polypeptide chains are subject to LLPS. The LLPS of cationic peptide-like polymers and nucleic acids proceeds via similar mechanisms, which has attracted attention in the context of investigating the origin of life, where it has been discussed in relation to the enrichment of substances (Poudyal et al., 2018) and the expression of catalytic activity during the primitive stages of the earth (Poudyal et al., 2019). Given the potential diverse involvement of DNA in current and primitive biological LLPS, it is crucial to understand which properties of DNA are most involved in the process in order to elucidate the molecular mechanisms of biological LLPS.

Liquid–liquid phase separation systems of DNA/cationic polypeptides have been used to investigate the contribution of physicochemical properties of DNA to phase separation. Examples include the use of poly-L-lysine and single- or double-stranded DNA, where differences in local flexibility, depending on DNA sequence and hybridization, have been shown to alter phase behavior (Shakya and King, 2018a; Vieregg et al., 2018). To gain insight into the contribution of the chemical structure of DNA in LLPS, here, we examined the role of the constituent components of DNA, i.e., a phosphate group, a deoxyribose sugar, and a nucleobase, in LLPS with cationic polypeptide chains. Here, linker histone H1 (H1), which is a key regulator of chromatin condensation, was chosen as a model polypeptide. Spectroscopic and microscopic observations after mixing H1 with single-stranded DNA (ssDNA)-based oligomers in which one or more of the constituent components of DNA were removed showed that in addition to the expected electrostatic interactions between the anionic phosphate groups of the oligomers and the cationic residues of H1, interactions involving nucleobases and deoxyriboses promoted the generation of LLPS, increased the density of ssDNA in the formed droplets, and simultaneously decreased their fluidity under low-salt conditions. Furthermore, we found that microscopically distinguishable non-spherical assemblies were formed in solutions containing relatively high concentrations of H1 for all the oligomers, and that these assemblies contained both liquid-like mobile and solid-like immobile fractions. This study is thus expected to provide a basic understanding of the phase behavior of DNA/cationic polypeptide mixtures, and may contribute to elucidate, for example, the mechanism governing LLPS-mediated formation of various intracellular biomolecular condensates containing DNA and RNA and the significance of the chemical structure of nucleic acids from an evolutionary perspective.

## Materials and Methods

### Materials

Tris-EDTA (TE) buffer [10 mM Tris-HCl; 1 mM EDTA; pH = 7.4] was obtained from Takara Bio Inc. (Shiga, Japan). Histone H1 (H1) from bovine thymus was obtained from Signal Chem (Richmond, BC, Canada). Prior to experiments, the buffer of the supplied H1 solution (1.0 mg/mL) was replaced with the TE buffer according to the previously reported procedure (Mimura et al., 2021). After the replacement, the concentration of H1 was determined from the absorbance at 280 nm using a NanoDrop One^*C*^ spectrophotometer (Thermo Fisher Scientific Inc., Waltham, MA, United States) and the molar extinction coefficient at 280 nm (4470 M^–1^ cm^–1^) calculated by the ProtParam tool. The oligo-deoxyribonucleotides with carboxyfluorescein (FAM) at the 3′-terminus and abasic oligo-deoxyribonucleotides with FAM at the 5′-terminus were synthesized by Eurofins Genomics (Ebersberg, Germany) and Nihon Gene Research Laboratories Inc. (Miyagi, Japan), respectively. The concentration of FAM-labeled oligomers was determined from the absorbance using the molar extinction coefficient of FAM at 490 nm (80,000 M^–1^ cm^–1^) in Tris-HCl buffer (pH = 9.0).

### Turbidity Measurements

Turbidity measurements were performed using a spectrophotometer (UV-1800; Shimadzu Co., Tokyo, Japan). A solution (150 μL) containing 5.0 μM oligomer in TE buffer was placed in a 1 cm quartz cell and the absorbance at 500 nm was recorded at 25°C. Subsequently, the absorbance was acquired consecutively after the addition of 0.2 μL aliquots of 300 μM H1 solution.

### Phase-Contrast Microscopy Imaging

To obtain the phase diagrams, solutions (20 μL) containing 1.0–10.0 μM oligomer and 1.0–10.0 μM H1 in TE buffer were prepared in the wells of a 384-well black clear-bottom microplate (Greiner Bio-One, Kremsmüster, Australia) using an Andrew+ liquid handling robot (Andrew Alliance SA, Geneva, Switzerland). After overnight incubation at room temperature with a lid to avoid evaporation, images of the solution were obtained using a phase-contrast microscope (Eclipse Ts2R, Nikon, Tokyo, Japan) equipped with a DS-Fi3 digital camera (Nikon, Tokyo, Japan). The process of fusion between the droplets was observed via time-lapse photography (increments: 0.1 ms) using a phase-contrast microscope after allowing solutions containing 5.0 μM oligomer and 2.5 or 7.5 μM H1 to incubate for 1 h at room temperature.

### Fluorescence Recovery After Photobleaching (FRAP) Measurements

Aliquots (50 μL) containing 10.0 μM FAM-labeled oligomer in TE buffer were mixed with solutions (50 μL) containing 5.0 μM or 15.0 μM H1 in TE buffer (pH = 7.4). After 4 h of incubation at room temperature, aliquots (50 μL) of the mixtures were placed on a cell-culture dish, and the samples were imaged using an FV1000 confocal microscope (Olympus Corp., Tokyo, Japan) at 60× with an excitation wavelength of 488 nm. The 488 nm laser was applied to the region of interest at full power for 1.0 s, and the fluorescence recovery was then recorded for 40 s. The fluorescence intensity of the photobleached area was normalized relative to that of the unbleached area and the background. The time course of the normalized fluorescence intensity was fitted to a single exponential function (eq. 1) in order to obtain the recovery time *τ.*

(1) $$f\left(t\right)=f_{0}+A\left(1-e^{-\frac{t}{\tau }}\right)$$

where, *f*_0_ is the fluorescence intensity at the time of photobleaching, A is an arbitrary constant, and *t* is the time after photobleaching.

## Results

### Experimental Design

**FIGURE 1:**
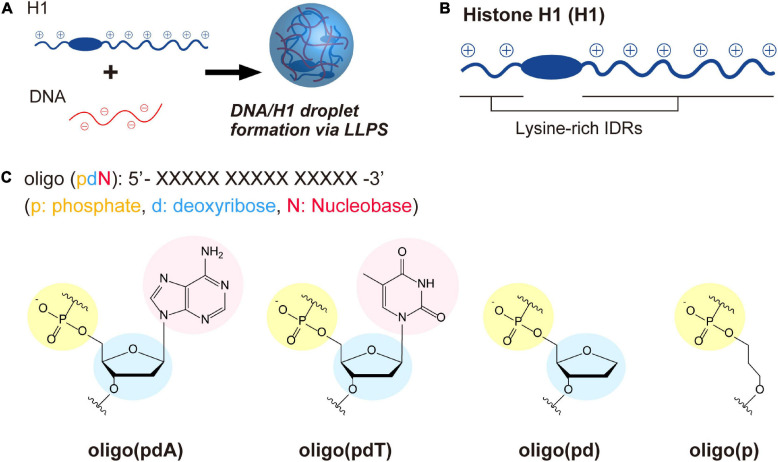
LLPS system and molecules used in this study. **(A)** Schematic illustration of droplet formation in ssDNA/H1 mixtures via LLPS. **(B)** Structural drawing of histone H1 with lysine-rich IDRs at the N- and C-terminus. The IDRs were estimated using three different structure-prediction algorithms (for details, see the Supplementary Figure 1). **(C)** Chemical structures of the ssDNA-based oligomers.

The role of each constituent components of DNA in LLPS was investigated through the interactions of ssDNA-based oligomers with cationic polypeptide chains. For this purpose, we used a model system in which liquid droplets are formed by mixing linker histone H1 (H1) and ssDNAs (Figure 1A; Shakya and King, 2018b; Turner et al., 2018; Mimura et al., 2021). H1 has a long and a short cationic lysine-rich intrinsically disordered region (IDR) at its C- and N-terminus, respectively (Figure 1B and Supplementary Figure 1). The C-terminal IDR has been proposed to play an important role in the organization of chromatin domains via a LLPS process involving interactions with inter-nucleosome linker DNAs (Gibson et al., 2019; Shakya et al., 2020). Four FAM-labeled 15-unit repeating oligomers consisting of different DNA components were selected as partners of H1 (Figure 1C): deoxyadenylic acid [oligo(pdA)], deoxythymidylic acid [oligo(pdT)], a deoxyribonucleotide lacking a nucleobase component [oligo(pd)], and a deoxyribonucleotide lacking both nucleobase and deoxyribose components [oligo(p)]. These oligomers do not exhibit any specific conformation and have the same total charge and chain length. Thus, we assumed that any differences in the LLPS of these oligomers with H1 would be due to factors other than electrostatic interactions between the positively charged residues of H1 and the negatively charged phosphate groups of the oligomers. Throughout this study, the effects of each component were examined by comparing spectroscopic and microscopic results after mixing H1 and these oligomers at physiological pH (pH = 7.4) under salt-free conditions to maintain the interactions underlying the phase separation.

### LLPS of Mixtures of the ssDNA-Based Oligomers and H1

**FIGURE 2:**
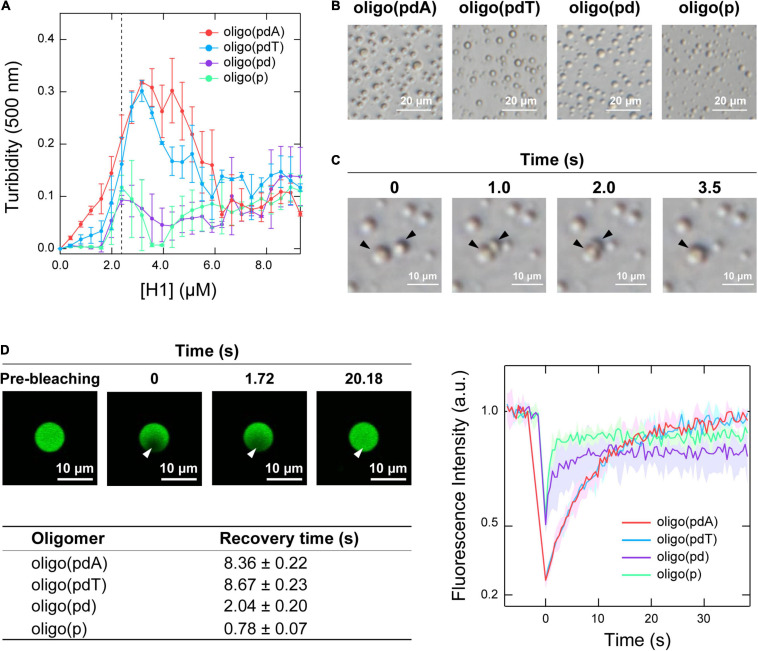
LLPS of mixtures of the oligomers and H1. **(A)** Turbidimetric titration curves of H1 against solutions containing 5.0 μM of the oligomers. **(B)** Phase-contrast microscopy images of the solutions under the conditions indicated by the dotted line in panel A (5.0 μM oligomer and 2.5 μM H1); scale bar = 20 μm. **(C)** Fusion process of the spherical assemblies formed by oligo(pdA) (5.0 μM) and H1 (2.5 μM); scale bar = 10 μm. **(D)** Left: confocal fluorescence microscopy images during FRAP measurements. White arrowhead indicates the bleached area; scale bar = 10 μm. Right: FRAP recovery curves for the oligomers; values shown represent mean values ± standard deviation (*n* = 3). The table below lists the fluorescence-recovery times calculated by single-exponential fitting. All experiments were carried out in 10 mM Tris-EDTA buffer (pH = 7.4).

Initially, the contribution of each DNA component to LLPS was examined by measuring the changes in the turbidity of mixed solutions of the oligomers and H1. When H1 was titrated against solutions containing each of the oligomers (5.0 μM), all the solutions became turbid with increasing H1 concentration, indicating that oligomer/H1 assemblies were formed in all cases (Figure 2A). The decreases in turbidity that were observed with further increasing the H1 concentration were due to the destabilization of the assemblies by the excess cationic charges, which is often observed in the assembly of oppositely charged polymers and is referred to as the ‘charge-inversion mechanism’ (Nguyen et al., 2000; Banerjee et al., 2017). The H1 concentration at which the maximum turbidity was observed varied slightly among the oligomers depending on whether they contained a nucleobase; this value was 3.0 μM for oligo(pdA) and oligo(pdT) and 2.5 μM for oligo(pd) and oligo(p). Interactions involving the nucleobases likely increased the number of H1 molecules that could bind to one oligomer, similarly to in a previous report (Chollakup et al., 2010). The two nucleobase-containing oligomers exhibited similar maximum turbidity values, i.e., oligo(pdA) and oligo(pdT) both showed maximum turbidities nearly three times greater than those of oligo(pd) and oligo(p), suggesting that the nucleobase moieties may have increased the amount of ssDNA/H1 assemblies. In addition, we found that the solution was not completely transparent in the presence of excess H1 (>6.0 μM); instead, the turbidity gradually increased in a concentration-dependent manner above the charge-inversion threshold (*vide infra*).

Phase-contrast microscopy images showed the formation of spherical assemblies in solutions containing 5.0 μM oligomer and 2.5 μM H1 (Figure 2B), i.e., at the H1 concentration where the oligomers without nucleobases exhibited their maximum turbidity (Figure 2A). Consistent with the order of the maximum turbidity values, the oligomers with nucleobases formed larger assemblies than those without (Supplementary Figure 2). These assemblies rapidly fused within seconds upon contact (Figure 2C and Supplementary Figure 3). This highly fluidic behavior indicates that these assemblies are liquid droplets formed via LLPS.

Confocal fluorescence microscopy images confirmed that all the FAM-labeled oligomers were concentrated within the droplet (Figure 2D and Supplementary Figure 4). Therefore, the motility of the oligomers within the droplets was compared using fluorescence recovery after photobleaching (FRAP) measurements (Taylor et al., 2019). The fluorescence intensity inside the droplet recovered within 30 s after photobleaching (Figure 2D and Supplementary Figure 4), indicating dynamic diffusion of the oligomers within the droplets. The fluorescence recovery time decreased with increasing removal of DNA components [oligo(pdT) ≈ oligo(pdA) > oligo(pd) > oligo(p)]. The density of oligomers inside the droplets followed the same order (Supplementary Figure 5). Therefore, both the nucleobase and deoxyribose moieties likely function as regulators in increasing the density and decreasing the motility of ssDNAs inside droplets.

**FIGURE 3:**
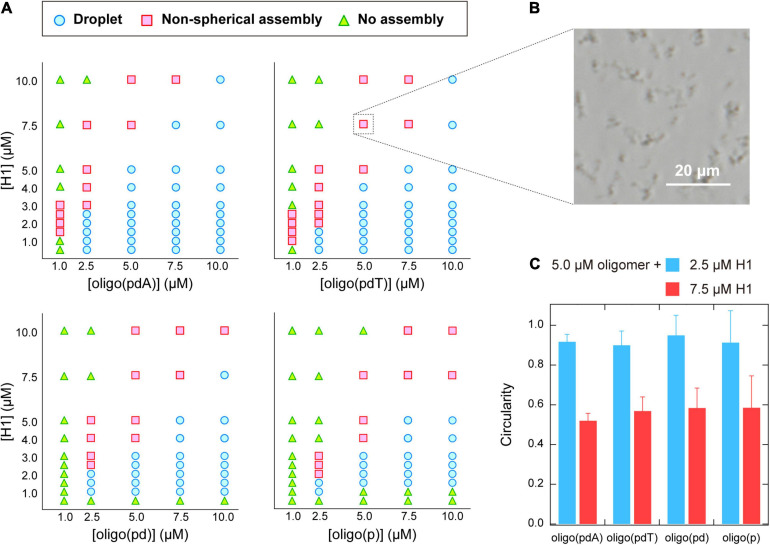
**(A)** Phase diagrams of aqueous mixtures of oligomers and H1. **(B)** Phase-contrast microscopy image of typical non-spherical assemblies; 5.0 μM poly(pdT) and 7.5 μM H1; scale bar = 20 μm. **(C)** Circularity of the assemblies formed in solutions containing 5.0 μM oligomer and 2.5 or 7.5 μM H1. The values were obtained by analyzing the fluorescence microscopy images of the solutions under the indicated conditions using the image processing software *image J*.

The phase diagrams for the concentrations of each molecule were constructed based on phase-contrast microscopy images to better understand the binary phase separation systems containing the various oligomers and H1 (Figure 3A). For all the oligomers, non-spherical assemblies were observed rather than the droplets, i.e., spherical assemblies, under some conditions. Both types of assemblies were the result of phase separation, but have been distinguished in the phase diagrams. Consistent with the turbidity measurements, the area of (i) LLPS and (ii) phase separation including spherical or non-spherical assemblies were larger for oligo(pdA) and oligo(pdT) than for oligo(pd) and oligo(p). The critical concentration of H1, i.e., the minimum molecular concentration required for LLPS, exhibited a similar trend. Of the oligomers without nucleobases, oligo(pd) appeared to exhibit a slightly lower critical concentration of H1. For instance, 5.0 μM oligo(pd) underwent LLPS with 1.0 μM H1, while oligo(p) did not under the same conditions. These results indicate that not only the nucleobase moieties, but also the deoxyribose moiety, promote phase separation with H1.

### Characterization of Non-spherical Assemblies Formed Between ssDNA-Based Oligomers and H1

The phase diagrams of the concentration of the oligomer vs. that of H1 revealed that non-spherical assemblies, rather than the spherical droplets (Figure 2B), were formed under certain solution conditions (Figure 3B). Interestingly, the non-spherical assemblies were only formed at relatively high concentrations of H1 for all the oligomers. For example, at the oligomer concentration used in the turbidity measurements (5.0 μM; Figure 2A), the boundaries for non-spherical assembly formation were approximately 7.5, 5.0, 4.0, and 4.0 μM of H1 for oligo(pdA), oligo(pdT), oligo(pd), and oligo(p), respectively (Figure 3A). Based on these results, the gradual increase after the decrease in turbidity at higher H1 concentrations (Figure 2A) was attributed to the transition from spherical droplets to non-spherical assemblies. It should be noted here that spherical droplets were not formed at an oligomer concentration of 1.0 μM for the oligomers with nucleobases.

More specifically, when relatively low (2.5 μM) or high (7.5 μM) concentrations of H1 were mixed with 5.0 μM oligomer solutions, the circularity of the assemblies differed significantly, with values of ∼0.9 and ∼0.5, respectively, for all oligomers (Figure 3C). Since assemblies formed via LLPS exhibit a spherical morphology (i.e., the circularity is close to 1.0) to minimize their surface energy, the observed non-spherical assemblies are generally regarded as ‘aggregates’ formed via so-called liquid–solid phase separation (Brangwynne et al., 2009; Yamasaki et al., 2020). Thus, the observed decrease in the circularity of the assemblies suggests a transition from liquid droplets to aggregates or gels. Such transitions have previously been observed in protein- (Patel et al., 2015; Matsuda et al., 2018) and DNA-related assemblies (Vieregg et al., 2018). As a related example, liquid droplets of the protein HP1α on heterochromatin domains decrease in circularity as the cell cycle progresses, and the internal molecules become immobile (Strom et al., 2017). In light of these phenomena, we further examined whether the observed non-spherical assemblies of the ssDNA-based oligomers and H1 also have aggregate-like properties.

**FIGURE 4:**
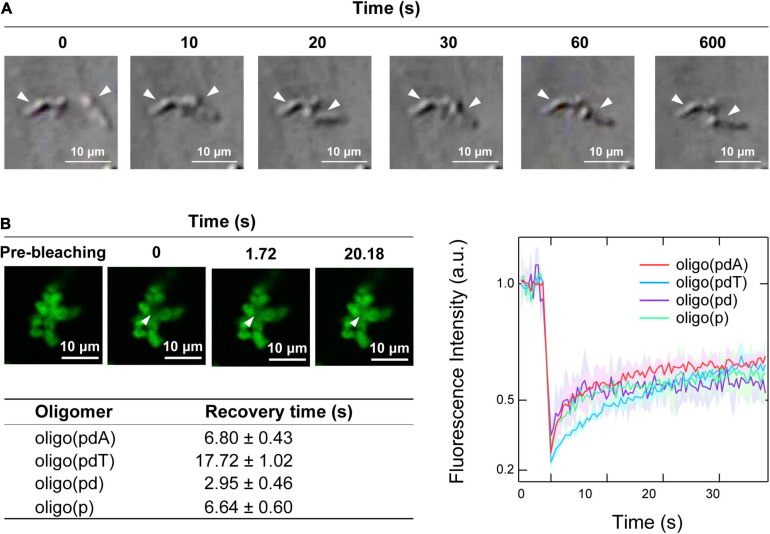
Physical properties of the non-spherical assemblies. **(A)** Collision process of the non-spherical assemblies formed by oligo(pdA) (5.0 μM) and H1 (7.5 μM); scale bar = 10 μm. **(B)** Left: confocal fluorescence microscopy images during the FRAP measurements. White arrowheads indicate the bleached areas; scale bar = 10 μm. Right: FRAP recovery curves for the oligomers; values shown represent mean values ± standard deviation (*n* = 3). The table below lists the fluorescence-recovery times calculated by single-exponential fitting.

Time-lapse images of the collision process of the non-spherical assemblies showed that none of the oligomer/H1 assemblies fused via so-called Ostwald ripening (Voorhees, 1992), but instead simply remained adsorbed while retaining their shape (Figure 4A and Supplementary Figure 6). There was therefore a possibility that the non-spherical assemblies had gel- or aggregate-like properties, unlike the spherical droplets, which can fuse rapidly (Figure 2C).

Confocal fluorescence microscopy images of the non-spherical assemblies revealed a cluster-like morphology, which consisted of many small near-spherical particulates (2–3 μm in diameter) adhered to one another (Figure 4B), as have been seen for protein–polyelectrolyte complexes or protein aggregates (Krebs et al., 2007; Cousin et al., 2011; Tomita et al., 2011). FRAP measurements were then carried out on these assemblies to investigate the fluidity of the internal oligomers. Unexpectedly, the fluorescence of the photobleached region of the assemblies was recovered over time (Figure 4B and Supplementary Figure 7). The degree of fluorescence recovery, i.e., the mobile fraction (Zhu et al., 2019), was about 50% regardless of the oligomer structure (Figure 4B). Interestingly, the calculated fluorescence recovery times were comparable to those of the spherical droplets (Figure 2D), although no clear trend with regard to the presence or absence of DNA components could be discerned (Figure 4B). These FRAP results suggest that a fraction of the oligomers inside the small particulates diffuse like in the spherical droplets; consistent with this result, the non-spherical assemblies dissolved upon adding salt (Supplementary Figure 8). The remaining immobile solid-like fraction may have prevented the fusion of the small particulates, but diffusion of the oligomers may be possible between the adhered particulates, as has been observed in protein–RNA condensates (Boeynaems et al., 2019).

## Discussion

In this study, we showed that the constituent components of DNA, i.e., nucleobase and deoxyribose moieties, as well as anionic phosphate groups, promote both liquid–liquid, and liquid–solid phase separation with cationic histone H1. When liquid droplets are formed, these moieties regulate the density and motility of the ssDNAs inside the droplet. In the following sections, we discuss the mechanisms by which phase separation occurs and the biological significance of the phase-separation state in terms of the DNA components.

### Interactions Between Each DNA Component and H1 in the Promotion of LLPS

A comparison of the four model oligomers with different DNA components but the same charge revealed that the nucleobase components markedly promoted LLPS with H1. Electrostatic interactions are the primary driving force for biological LLPS (Boyko et al., 2019), including that of the duplex DNA/H1 system (Turner et al., 2018); however, interactions related to π-electrons are also significant. For example, π–π interactions also play a major role in the formation of various biomolecular condensates (Chong et al., 2018; Vernon et al., 2018). It is therefore plausible that π–π interactions between the aromatic amino acids of H1 (3 Tyr and 2 Phe in 194 aa; for the sequence of H1, see Supplementary Figure 1) and the nucleobases and/or those between nucleobases promoted LLPS, as has been previously suggested (Mimura et al., 2021). Cation–π interactions may also be involved (Chong et al., 2018; Qamar et al., 2018). H1 contains 55 Lys and 7 Arg residues. Although cation–π interactions related to biological LLPS are often mediated by Arg, Lys can also promote LLPS with nucleobases via cation–π interactions (Biot et al., 2002; Shakya and King, 2018b). These π-electron-related interactions should be responsible for the high LLPS potential of the nucleobase components of DNA.

The introduction of the deoxyribose moiety into the phosphate groups of oligo(p) also slightly promoted phase separation with H1 (Figure 3). The deoxyribose in DNA tends to exhibit van der Waals interactions with amino acids such as Lys, Thr, and Ser (Luscombe et al., 2001), which are abundant in H1. In addition, sugar–π interactions between deoxyribose sugars and amino acids are found in the crystal structures of protein–DNA complexes (Wilson et al., 2016), and thus, this type of interactions could potentially promote the LLPS of DNA/H1 mixtures. Another possibility is that since changes in the flexibility of DNA contribute entropically to LLPS with cationic polymers (Shakya and King, 2018a; Vieregg et al., 2018), an increase in DNA rigidity due to the introduction of the deoxyribose moieties may also be involved.

### Physical Properties of the Assemblies and Their Possible Relevance to Biological Events

In the phase-separation systems of the DNA-based oligomers and H1, spherical droplets were formed via LLPS when the concentration of H1 was relatively low compared to that of the oligomers, while relatively high concentrations of H1 induced the formation of non-spherical assemblies. When droplets were formed, the presence of nucleobase and deoxyribose moieties increased the density and decreased the diffusion rate of the oligomers inside the droplets (Figure 2 and Supplementary Figure 5). These were correlated with the effect of the moieties on the critical concentration at which LLPS occurs (Figure 3).

The non-spherical assemblies are more complicated. The cluster-like assemblies, which were formed via the adhesion of small near-spherical particulates, did not undergo fusion upon contact (Figure 4A); similar phenomena have been observed in chromatin (Li et al., 2021) and RNA condensates with gel-like properties (Roden and Gladfelter, 2021). Although these assemblies appear to exhibit solid-like properties, FRAP measurements demonstrated that they consist in part of liquid-like mobile fractions (Figure 4B). The cell nucleus contains loosely condensed euchromatin regions where gene transcription is active and highly condensed heterochromatin regions where it is inactive. As the depletion of H1 triggers chromatin de-condensation in the nucleus, H1 is thought to play an integral role in heterochromatin formation (Izzo and Schneider, 2016; Izquierdo-Bouldstridge et al., 2017). The addition of H1 to nucleosome array droplets reduces their fluidity, resulting in heterochromatin-like properties (Gibson et al., 2019). Taking these facts into account, H1 may form immobilized fractions by reducing the fluidity of DNA-containing droplets in a concentration-dependent manner, as observed in this study. The IDR at the N-terminus of H1 does not have a high affinity for chromatin, but does toward heterochromatin proteins (Daujat et al., 2005; Sarg et al., 2015). This difference in affinity may cause local deviations in the concentration of H1 in the nucleus and subsequent interaction of the C-terminal IDR with DNA, possibly contributing to the formation of cluster-like heterochromatin.

The results of the present study suggest that the constituent components of DNA may commonly promote phase separation with cationic polypeptide chains related to biological LLPS. However, in general, the experiments of this study were performed under low-ionic-strength conditions to facilitate the observation of phase separation. Therefore, it could be argued that evidence is still insufficient to support a strong case for a direct relationship with biological events as described above. Although electrostatic interactions are weakened at high ionic strength, which often leads to a dissolution of the liquid droplets, phase separation can occur if the concentrations of DNA and proteins are sufficiently high or if a crowded environment, such as the inside of a cell, is reproduced (André and Spruijt, 2020). Under conditions where such electrostatic interactions are suppressed, the contribution of uncharged components revealed in this study may become high in comparison. Nevertheless, the states of phase separation, such as the droplets or non-spherical assemblies, should be governed by the balance of interactions, and therefore, studies under conditions closer to the physiological environment should be conducted to provide a more accurate understanding.

Chemical modifications of biomolecules involved in the epigenetics, such as post-translational modifications (Aumiller and Keating, 2016; Gibson et al., 2019) and DNA modifications (Wang et al., 2020), have been shown to regulate the biological LLPS and prebiotic-relevant coacervation (Ukmar-Godec et al., 2019). The strategies to systematically design constituent units through chemical approaches can be expected to provide valuable insights into the complicated phase-separation mechanisms of biomacromolecules, which are polymer chains of amino acids and nucleic acids composed of diverse elements.

## Conclusion

In summary, our study using single-stranded DNA-based oligomers in which one or more of the constituent DNA components had been removed revealed that the nucleobase and deoxyribose DNA moieties (i) promote the LLPS with H1 and (ii) control the motility and density of the oligomers inside the droplets under low-salt conditions. In addition, we observed the formation of non-spherical assemblies with mobile and immobile fractions when the concentration of H1 was higher than that of the oligomers, which could potentially be related to the role of H1 in chromatin organization. This study may thus provide fundamental insight into the formation mechanisms of various biomolecular condensates containing nucleic acids, such as chromatin, and may also contribute to the understanding of biomacromolecules in the context of the origin of life.

## Data Availability Statement

The original contributions presented in the study are included in the article/Supplementary Material, further inquiries can be directed to the corresponding authors.

## Author Contributions

MM, ST, HS, and RK conceived and designed the experiments. MM, YS, and SI performed the experiments and analyzed the data obtained. MM, ST, HS, YS, and RK wrote and edited the manuscript. All authors contributed to the article and approved the submitted version.

## Conflict of Interest

The authors declare that the research was conducted in the absence of any commercial or financial relationships that could be construed as a potential conflict of interest.

## Publisher’s Note

All claims expressed in this article are solely those of the authors and do not necessarily represent those of their affiliated organizations, or those of the publisher, the editors and the reviewers. Any product that may be evaluated in this article, or claim that may be made by its manufacturer, is not guaranteed or endorsed by the publisher.
