# Supplementary material for: Uncharged Components of Single-Stranded DNA Modulate Liquid–Liquid Phase Separation With Cationic Linker Histone H1
Source: Front Cell Dev Biol. 2021 Aug 4;9:710729. doi: 10.3389/fcell.2021.710729 (PMC8371396; doi:10.3389/fcell.2021.710729)
Supplement: Supplementary file 1 [file Data_Sheet_1.PDF]

Supplementary Material

Supplementary Figures

(A) Sequence of H1

MTENSTSTPA AKPKRAKASK KSTDHPKYSD MIVAAIQAEK NRAGSSRQSI
QKYIKSHYKV GENADSQIKL SIKRLVTTGV LKQTKGVGAS GSFRLAKSDE
PKRSVAFKKT KKEVKKVATP KKAAKPKKAA SKAPSKKPKA TPVKKAKKKP
AATPKKTKKP KTVKAKPVKA SKPKKTKPVK PKAKSSAKRT GKKK

(B) Disorder prediction

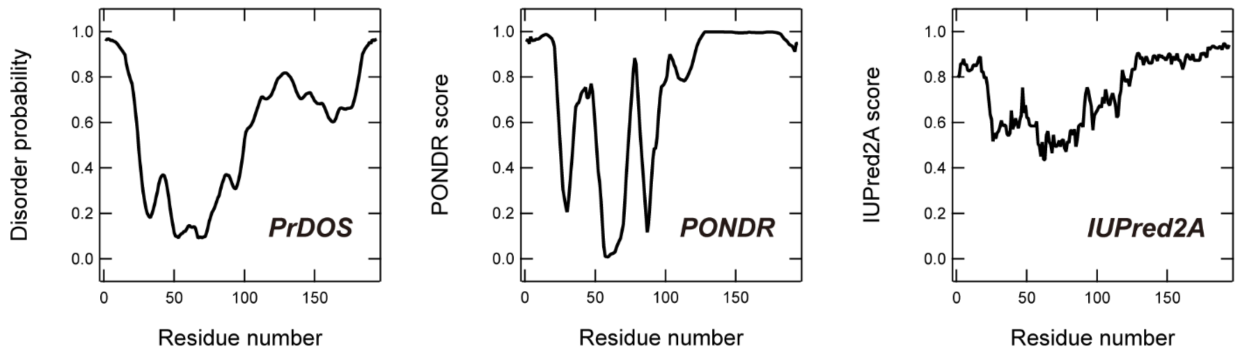

Supplementary Figure 1. Sequence and estimated structure of histone H1 (H1). (A) Sequence of H1 from bovine thymus obtained from Uniprot code Q0IIJ2. (B) Disorder probability of the H1 sequence as predicted using (left) the Protein DisOrder prediction System (PrDOS) (Ishida and Kinoshita, 2007), (middle) Predictor of Natural Disordered Regions (PONDR) (Linding et al., 2003), and (right) IUPred2A (Mészáros et al., 2018). Regions of the sequence that exhibit a score greater than 0.5 are defined as intrinsically disordered regions.

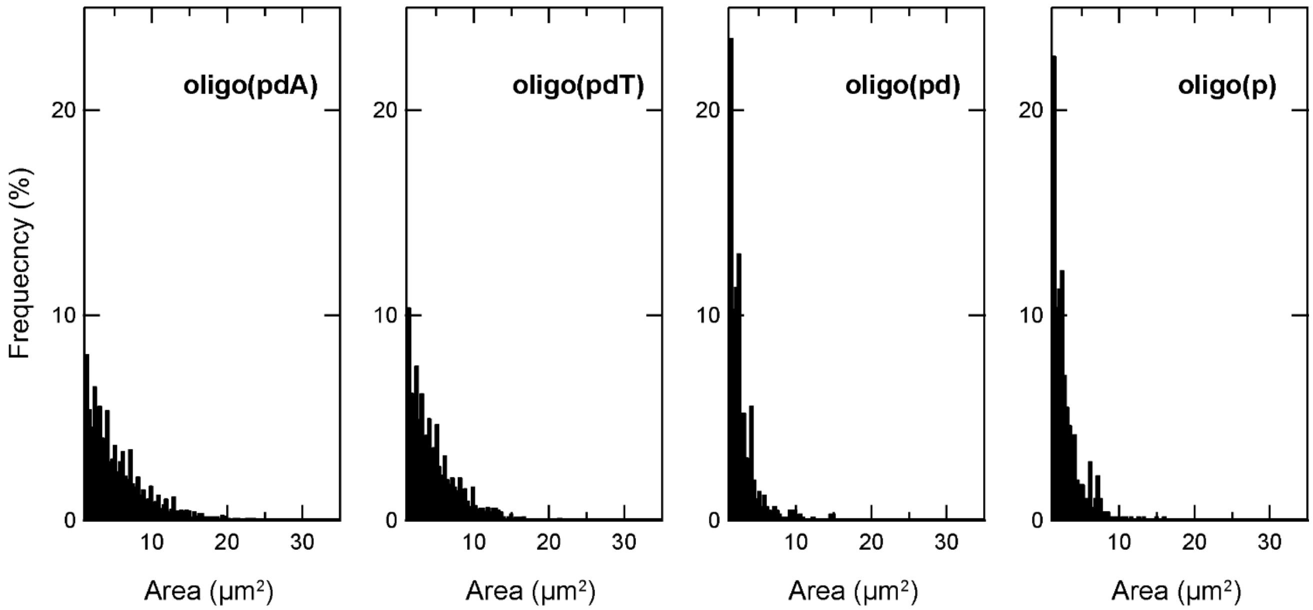

Supplementary Figure 2. Size distribution of the spherical assemblies formed in the solutions containing the oligomers (5 μM) and H1 (2.5 μM). Values were obtained by analyzing the fluorescence-microscopy images using the image-processing software *image J*.

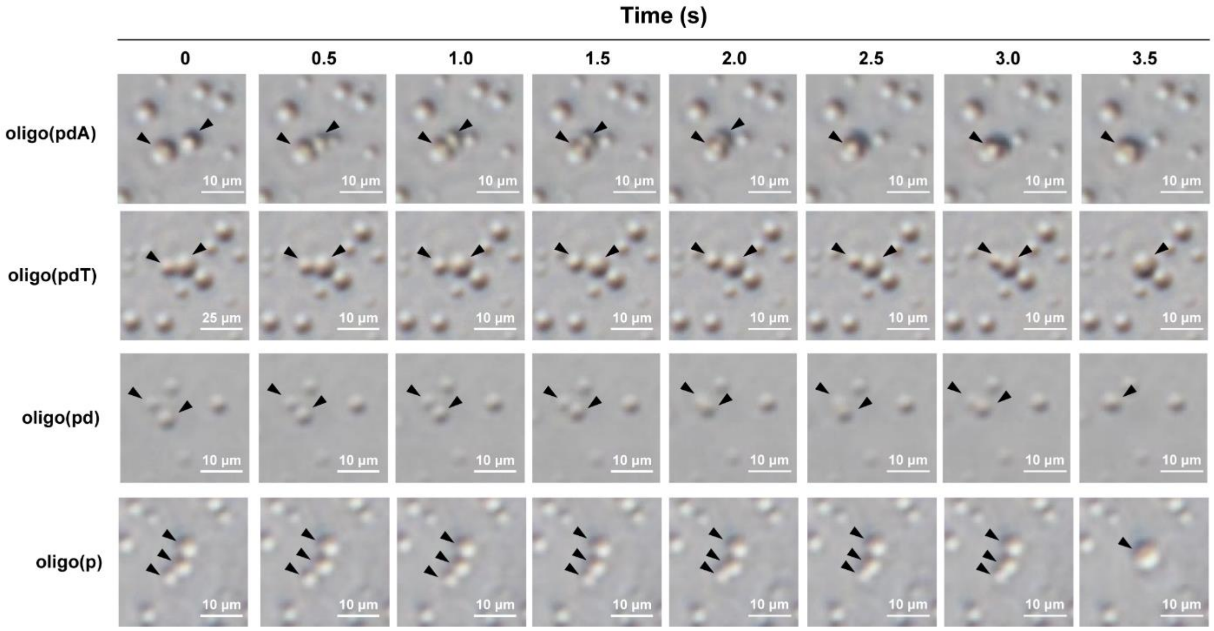

Supplementary Figure 3. Fusion processes of the spherical assemblies formed in the solutions containing the oligomers (5 μM) and H1 (2.5 μM) observed using a phase-contrast microscope; scale bar = 10 μm.

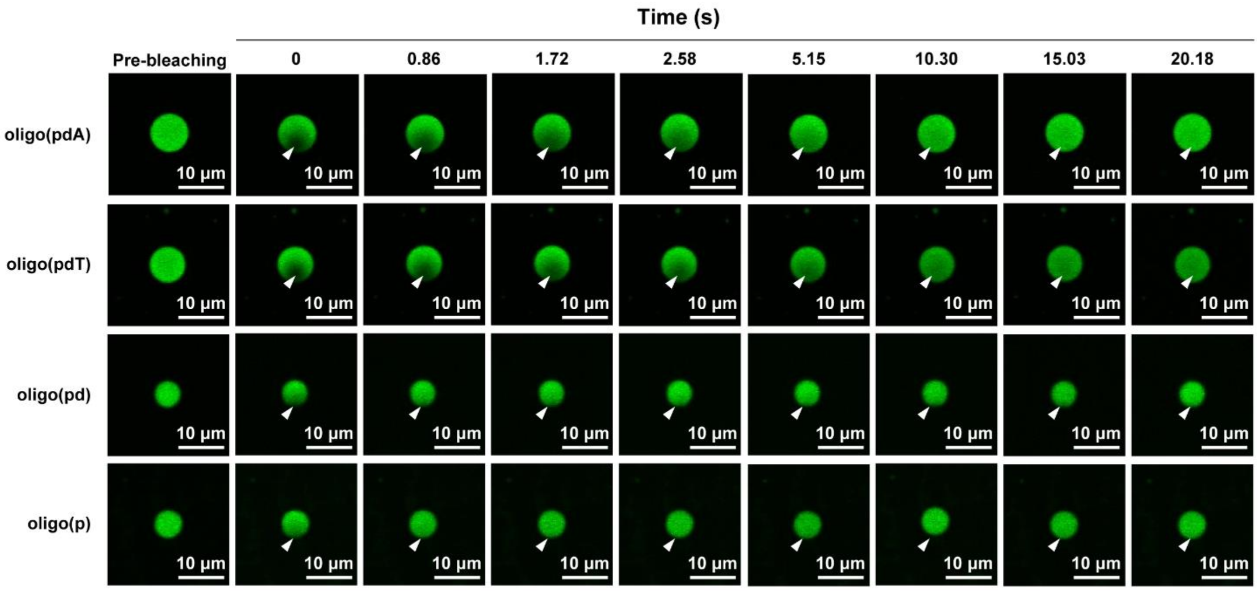

Supplementary Figure 4. Confocal fluorescence microscopy images during the FRAP experiments corresponding to Figure 2D; scale bar = 10 μm. White arrowheads indicate the bleached area.

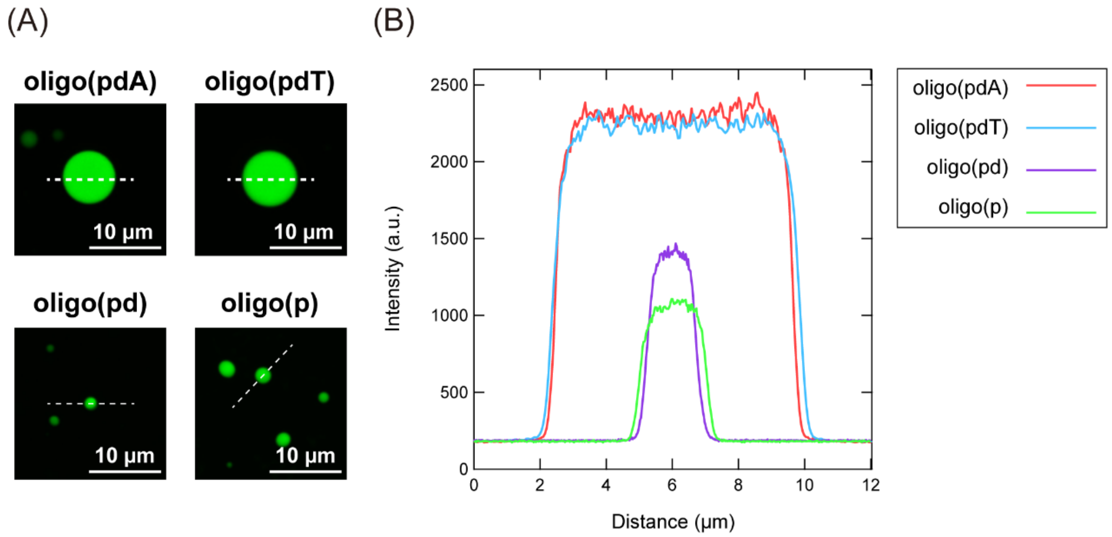

Supplementary Figure 5. Distribution of the oligomers inside and outside the droplets. (A) Confocal fluorescence microscopy images of the droplets formed by the oligomers (5 μM) and H1 (2.5 μM); scale bar = 10 μm. (B) Fluorescence intensities along the white dashed lines in each image in (A).

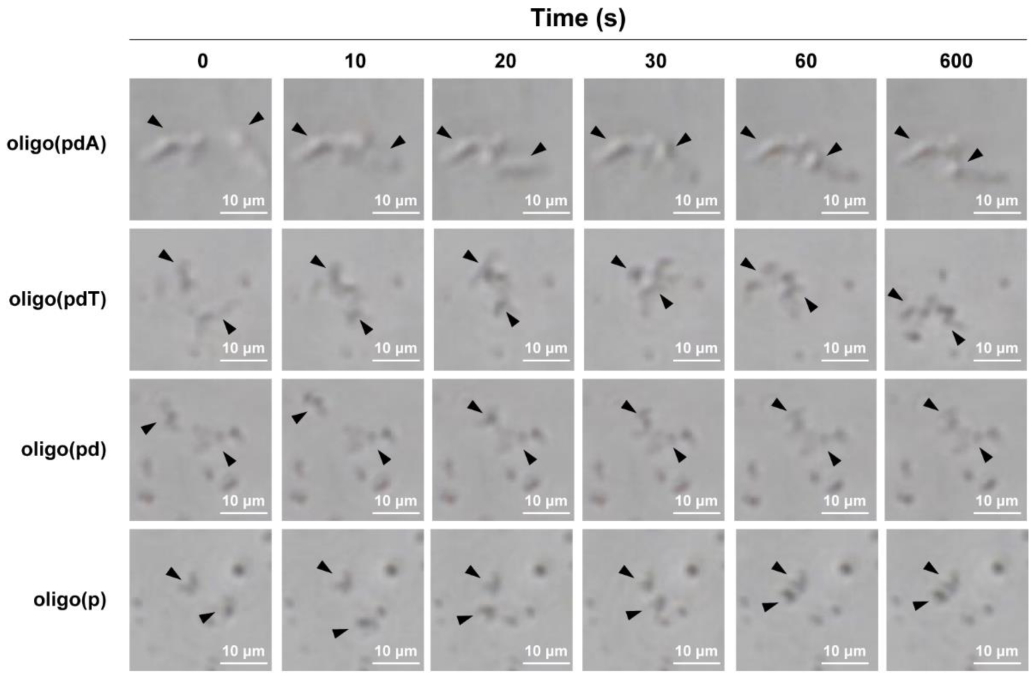

Supplementary Figure 6. Fusion processes of the non-spherical assemblies formed in the solutions containing oligomers (5 µM) and H1 (7.5 µM) observed by phase-contrast microscopy; scale bar = 10 µm.

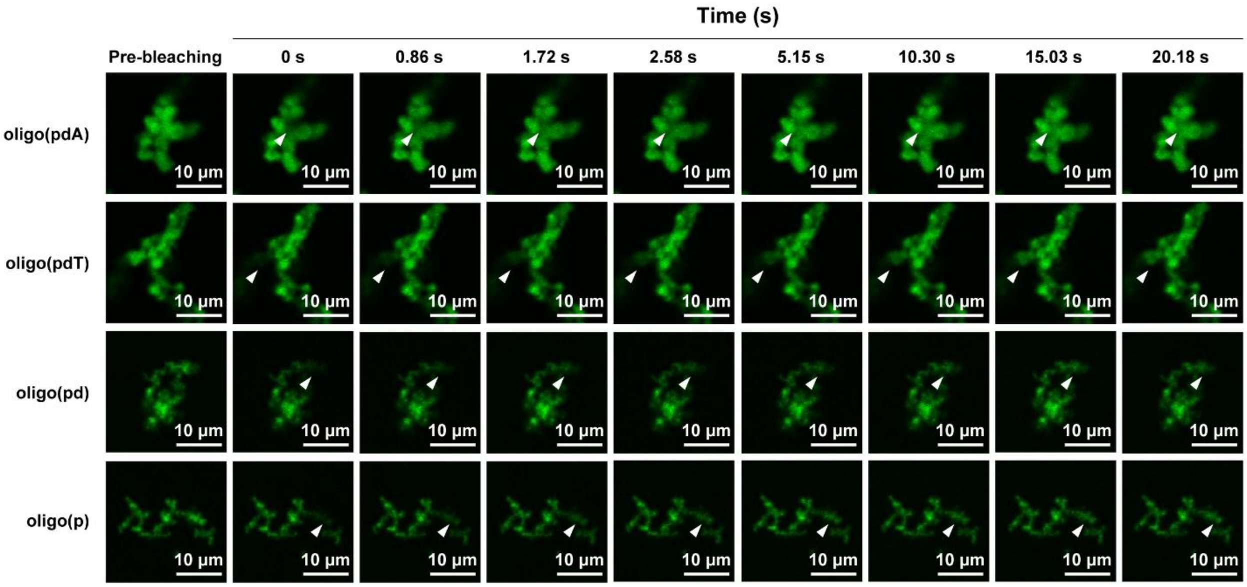

Supplementary Figure 7. Confocal fluorescence microscopy images during the FRAP experiments corresponding to Figure 4B; scale bar = 10 µm. White arrowheads indicate the bleached area.

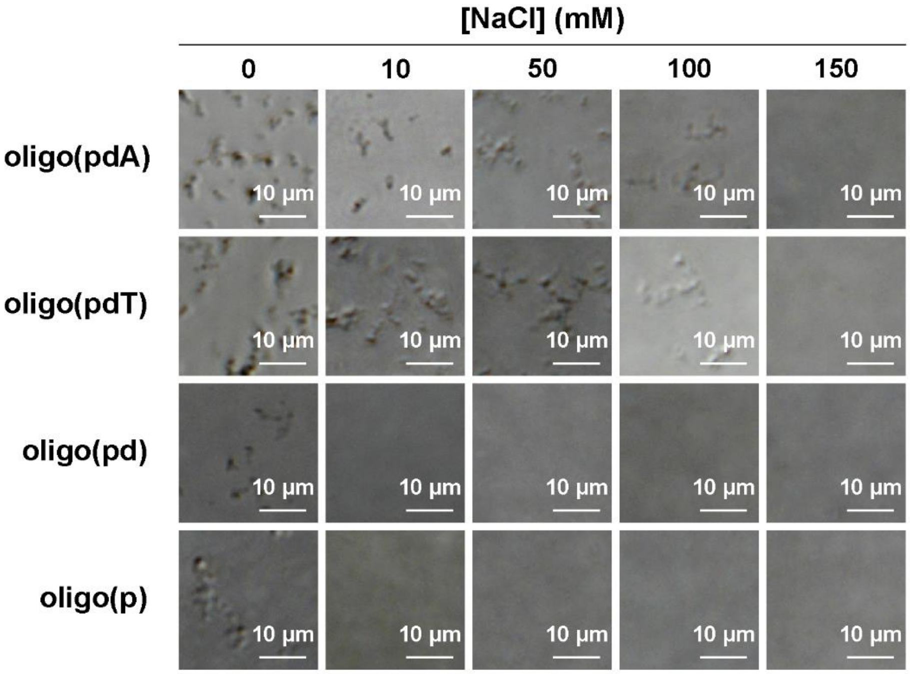

Supplementary Figure 8. Effect of adding NaCl (0-150 mM) on the formation/dissolution of non-spherical assemblies in the solutions containing oligomers (5.0 μM) and H1 (7.5 μM) observed by phase-contrast microscopy; scale bar = 10 μm.

References

Ishida, T., and Kinoshita, K. (2007). PrDOS: prediction of disordered protein regions from amino acid sequence. *Nucleic Acids Res.* 35, W460–464.

Linding, R., Jensen, L. J., Diella, F., Bork, P., Gibson, T. J., and Russell, R. B. (2003). Protein disorder prediction: implications for structural proteomics. *Structure* 11, 1453–1459.

Mészáros, B., Erdős, G., and Dosztányi, Z. (2018). IUPred2A: context-dependent prediction of protein disorder as a function of redox state and protein binding. *Nucleic Acids Res.* 46, W329–W337.
